# Unexpected bilateral massive pulmonary embolism

**DOI:** 10.1186/1865-1380-4-70

**Published:** 2011-11-18

**Authors:** Zaffer Qasim

**Affiliations:** 1 Emergency Department, Manchester Royal Infirmary, Oxford Road, Manchester M13 9WL, UK

## Case report

**Figure 1:**
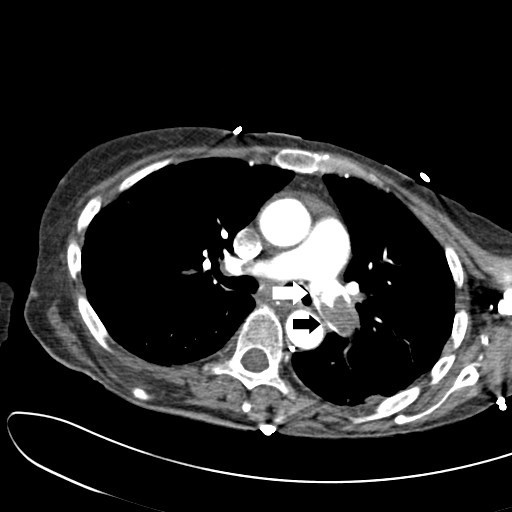
**CTPA image of left pulmonary artery showing saddle embolus (*black arrows*)**.

**Figure 2:**
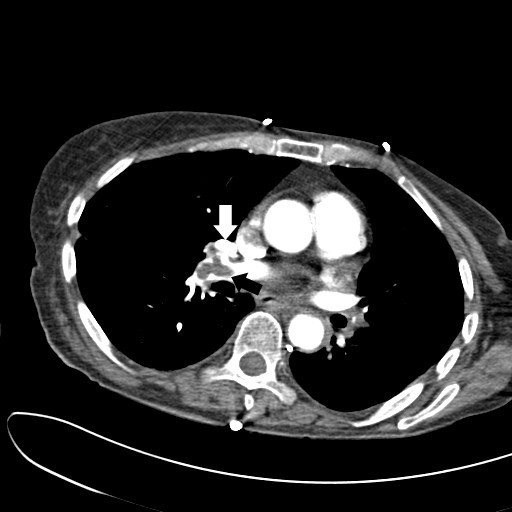
**CTPA image showing similar saddle embolus (*white arrows*) in right pulmonary artery of the same patient**.

A 59-year-old woman with a past history of rheumatoid arthritis arrived in our Emergency Department via ambulance. Her husband stated she had suddenly "appeared very strange" whilst preparing to go out for the afternoon, but could not identify specific symptoms. Physical examination showed her to have sinus tachycardia and tachypnea, but little else of note. Her oxygen saturations however rapidly dropped when she was taken off high-flow oxygen. Her D-dimer assay was markedly elevated, and urgent computed tomographic pulmonary angiography (CTPA) was performed (Figures 1 and 2). This showed large emboli (black arrows) in both the left (Figure 1) and right (Figure 2) pulmonary arteries (white arrows), with a saddle embolus noted on the right. Following the CTPA, she developed signs and symptoms of obstructive shock, requiring urgent thrombolysis using tenectaplase and admission to the intensive care unit. Her hospital stay was complicated by a lower respiratory tract infection, but she was discharged 17 days after her admission.

Rheumatoid arthritis may be complicated by venous thrombotic disease with up to 33% of cases being associated with antiphospholipid syndrome [1]. Antiphospholipid antibodies may have precipitated the events in our patient. When the patient's condition deteriorated, we resorted to thrombolytic therapy. There are clear indications for the administration of thromobolytic agents. The most recent recommendations from the American College of Chest Physicians [2] advise its use with evidence of hemodynamic compromise in the absence of contraindications to therapy, ideally via a peripheral vein, and utilizing a regimen with a short infusion time. There is less robust evidence to support the use of thrombolytics for high-risk, normotensive patients assessed to have a low bleeding risk, but outside these conditions, thrombolytics are not recommended.

## Competing interests

The author declares no competing interests.
